# Multimodal MRI accurately identifies amyloid status in unbalanced cohorts in Alzheimer’s disease continuum

**DOI:** 10.1162/netn_a_00423

**Published:** 2025-03-20

**Authors:** Giorgio Dolci, Charles A. Ellis, Federica Cruciani, Lorenza Brusini, Anees Abrol, Ilaria Boscolo Galazzo, Gloria Menegaz, Vince D. Calhoun, for the Alzheimer’s Disease Neuroimaging Initiative

**Affiliations:** Department of Computer Science, University of Verona, Verona, Italy; Department of Engineering for Innovation Medicine, University of Verona, Verona, Italy; Tri-Institutional Center for Translational Research in Neuroimaging and Data Science (TReNDS), Georgia State University, Georgia Institute of Technology, Emory University, Atlanta, GA, USA

**Keywords:** Alzheimer’s disease, Amyloid-beta, Connectivity, Deep learning, Explainable AI

## Abstract

Amyloid-*β* (A*β*) plaques in conjunction with hyperphosphorylated tau proteins in the form of neurofibrillary tangles are the two neuropathological hallmarks of Alzheimer’s disease. It is well-known that the identification of individuals with A*β* positivity could enable early diagnosis. In this work, we aim at capturing the A*β* positivity status in an unbalanced cohort enclosing subjects at different disease stages, exploiting the underlying structural and connectivity disease-induced modulations as revealed by structural, functional, and diffusion MRI. Of note, due to the unbalanced cohort, the outcomes may be guided by those factors rather than amyloid accumulation. The partial views provided by each modality are integrated in the model, allowing to take full advantage of their complementarity in encoding the effects of the A*β* accumulation, leading to an accuracy of 0.762 ± 0.04. The specificity of the information brought by each modality is assessed by post hoc explainability analysis (guided backpropagation), highlighting the underlying structural and functional changes. Noteworthy, well-established biomarker key regions related to A*β* deposition could be identified by all modalities, including the hippocampus, thalamus, precuneus, and cingulate gyrus, witnessing in favor of the reliability of the method as well as its potential in shedding light on modality-specific possibly unknown A*β* deposition signatures.

## Author Summary

In this work, we employed a multimodal MRI-based deep learning framework for the classification of unbalanced cohorts relying on the amyloid-*β* status in the Alzheimer’s disease continuum. To this end, structural, functional, and diffusion MRI data were used to feed a 3D-convolutional neural network and two different graph neural networks, respectively, reaching an accuracy of 0.762 ± 0.04. Post hoc explainability analysis was performed to extract the most relevant regions that led to the outcome, highlighting the involvement of different cortical and subcortical regions. This work provides evidence of the added value brought by exploiting different imaging modalities in decrypting the nature and extent of brain alterations in the amyloid-guided classification outcome.

## INTRODUCTION

The amyloid cascade hypothesis in Alzheimer’s disease (AD) posits that the accumulation of extracellular amyloid-*β* (A*β*) neuritic plaques in the brain leads to tauopathy and consequent neurodegeneration (Haass & Selkoe, 2022). Hence, A*β* deposition in the brain is considered to be the first step and the principal trigger of AD pathology (Fernandez-Alvarez, Atienza, & Cantero, 2023; Haass & Selkoe, 2022). In consequence, the neurodegeneration of gray matter (GM) may be related to the deposition of A*β* plaques, resulting in cerebral atrophy and synaptic loss (Serrano-Pozo, Frosch, Masliah, & Hyman, 2011), altering many brain regions, especially subcortical areas, and functional networks. Additionally, A*β* plaques in AD are also linked with demyelination of white matter (WM) tracts (Sánchez et al., 2020).

In clinical practice, the precise identification of A*β* proteins, and thus the classification of patients as A*β* positive or negative, is performed through amyloid positron emission tomography (PET) scans and cerebrospinal fluid (CSF) tests. Despite their undeniable utility, their use has some inherent limitations. First, limiting to one modality hides informative features that would require other kinds of investigation tools to be revealed. Then, amyloid PET uses ionizing radiation and radioactive tracers, is expensive, and is not widely available (Hansson et al., 2018; Lee, Kang, Jeong, & Kang, 2021). Additionally, the CSF test requires an invasive lumbar puncture.

A wider perspective on the disease can be obtained by adding views obtained by other imaging modalities. Among these, noninvasive MRI techniques are at the top of the list, allowing to assess structural and functional changes encoding the effect of the amyloid accumulation that can profitably be used as additional biomarkers. Structural MRI (sMRI) has been widely used for AD detection and early prediction due to its ability to detect GM atrophy and structural changes. Resting-state functional MRI (rs-fMRI) detects changes in blood oxygenation level-dependent signals, which depend on neurovascular coupling, and hence, indirectly measures brain neural activity (Johnson, Fox, Sperling, & Klunk, 2012). Different resting-state networks (RSNs), like the saliency and default-mode (DM) networks, have been shown to be altered in AD pathology (Palmqvist et al., 2017; Pini et al., 2021; Zhou et al., 2010). Finally, diffusion MRI (dMRI) is an imaging technique that relies on the movement of water molecules, allowing the assessment of both microstructural (Zucchelli et al., 2016) and structural connectivity (SC) changes (Sánchez et al., 2020). These two modalities are generally used to map the whole-brain connectomes, with rs-fMRI describing the functional connectivity (FC) between region pairs (usually in terms of correlations) and dMRI describing the SC (most often relying on the number of WM fibers linking the target regions).

Different studies focusing on A*β* classification tasks (positive vs. negative) in AD research have employed PET images and related features with deep learning (DL) models, while only a few works used MRI data. In Son et al. (2020), a slice-level approach for the identification of the A*β* status was considered in conjunction with a 2D-convolutional neural network (CNN) for feature extraction and classification. In the same manner, Reith, Koran, Davidzon, Zaharchuk, et al. (2020) adopted the slice-level approach for the classification of A*β* status employing two different 2D-CNNs (ResNet-50 and ResNet-152), both reaching high accuracy (around 0.95). On the other hand, Lee et al. (2021) considered three different well-known 3D-CNNs for detecting A*β* positivity, relying on 3D Florbetaben brain PET images. Kim et al. (2021) developed a particular CNN composed of different submodules for analyzing the 3D fluorodeoxyglucose (FDG)-PET images, converting them into slices following the three different axes. H. Kang and Kang (2023) faced the problem of A*β* classification relying on both early and delay-phase FBB PET images and tested their models considering both single and fused modalities. Using FDG and amyloid PET-derived images, they were able to achieve competitive performance in this task, with accuracies of around 0.80. More recently, Rasi, Guvenis, et al. (2024) employed FDG-PET-derived features in order to predict the A*β* status in the AD continuum. To this end, they tested eight different feature selection methods and eight different classifiers. Least absolute shrinkage and selection operator (LASSO) in conjunction with the Gaussian Naive Bayes (GNB) model performed better with respect to the others, achieving an area under the curve (AUC) of 0.924. Regarding MRI-based approaches for A*β* detection, Chattopadhyay et al. (2023) employed sMRI images in conjunction with a 3D-CNN, while Yang, Wu, Thompson, and Wang (2021) used an signed distance field (SDF)-based convolutional network to analyze the hippocampus region. Using sMRI-derived images and features, they were able to reach accuracies around 0.75. Due to the heterogeneous factors that lead to AD, in recent years, many studies have focused on multimodal DL models due to their ability to integrate information of different nature and to outperform single-modality methods (Abrol, Fu, Du, & Calhoun, 2019; Chattopadhyay et al., 2023; Dolci et al., 2023). Recently, graph neural and convolutional networks (GNNs/GCNs) have become popular in neuroscience due to their perfect fit for functional and structural brain networks. Cui et al. (2023) proposed a benchmark for analyzing fMRI and dMRI networks through GNNs, testing different messages passing, node features, and pooling operations, while Wee et al. (2019) developed a GCN to study the cortical thickness.

Although DL models can achieve high performance in different tasks, they do not easily provide interpretable output for what they have learned, which is particularly problematic in clinical and biomedical domains. To address this issue, eXplainable Artificial Intelligence (XAI) methods have been developed, allowing to identify the contributions of input features to final predictions, potentially highlighting crucial information for AD (Abrol et al., 2020, 2021; Böhle, Eitel, Weygandt, & Ritter, 2019; El-Sappagh, Alonso, Islam, Sultan, & Kwak, 2021).

In this study, the overall goal of the proposed approach consists of decrypting the signatures induced by A*β* accumulation in the views provided by s/rs-f/dMRI, taking advantage of their complementarity for capturing the A*β* status while exploiting their interplay for the identification of the regions that played a prominent role in determining the outcome. To instantiate this idea, we present a multimodal and explainable DL framework for the classification of A*β*-positive and A*β*-negative status relying on an unbalanced cohort of individuals spanning the AD continuum. The proposed framework includes structural and functional connectomes derived from dMRI and rs-fMRI, respectively, along with sMRI-derived GM 3D volumes in order to investigate complementary aspects as well as their relations. For the sake of the interpretability of the results, an extensive post hoc XAI analysis was then performed, pointing the spotlight on the input features that most influenced the final outcome.

## MATERIALS AND METHODS

### Dataset

The sMRI, rs-fMRI, and dMRI neuroimaging data used in the preparation of this article were obtained from the Alzheimer’s Disease Neuroimaging Initiative (ADNI) database (adni.loni.usc.edu). The ADNI was launched in 2003 as a public-private partnership, led by Principal Investigator Michael W. Weiner, MD. The primary goal of ADNI has been to test whether serial MRI, PET, other biological markers, and clinical and neuropsychological assessment could be combined to measure the progression of mild cognitive impairment (MCI) and early AD. For up-to-date information, please refer to www.adni-info.org.

One of the key strengths of the dataset is the inclusion/exclusion criteria adopted to recruit the subjects; subjects with neurological diseases other than AD and with different substance/drug use were excluded from the study. Due to this, the results we uncover related to A*β* are unlikely to be related to other diseases or the use of a particular substance. For more information about exclusion criteria, please refer to the official document at this link: https://adni.loni.usc.edu/wp-content/themes/freshnews-dev-v2/documents/consentForms/ADNI3_ProtocolVersion3.1_20201204.pdf.

In this work, our dataset was initially composed of 18,416 preprocessed sMRI images (from 2,144 subjects) from the ADNI 1, 2, 3, and GO phases, out of which 18,334 passed quality control (QC) (from 2,143 subjects). For rs-fMRI, 2,584 preprocessed images were considered (from 1,143 individuals) from the ADNI 2, 3, and GO phases, out of which 2,450 passed QC (from 1,105 individuals). For dMRI, 901 preprocessed images (from 901 subjects) from ADNI 3 were considered, out of which 894 (from 894 subjects) passed QC. Additional information about QC is detailed in the next paragraph. The images of the first available timepoint from only subjects belonging to control (CN), significant memory concern (SMC), early MCI (EMCI), late MCI (LMCI), and AD clinical classes, which had all three modalities, and available A*β* status were included. Lumbar puncture to retrieve CSF samples was performed using the procedures described on the ADNI website, and subjects were labeled as A*β* positive (POS) or negative (NEG) based on the A*β* protein levels reported by the CSF test. Similarly to Hansson et al. (2018), a cutoff of 980 pg/ml was used to define the A*β* status (i.e., < 980 pg/ml for positivity).

Aiming at the classification based on the A*β* status as the target outcome, the considered group of individuals was further split, gathering the A*β*-negative CN, SMC, and EMCI individuals in the NEG class (69, 75, and 41 subjects) and the A*β*-positive EMCI, LMCI, and AD in the POS class (53, 53, and 27 subjects), respectively, resulting in an unbalanced data split with respect to the disease stage. The LMCI and AD groups were not included in the A*β*-negative class since they could represent different underlying conditions linked with the functional decline (e.g., Lewy body dementia, frontotemporal dementia, vascular dementia, TAR DNA-binding protein 43 pathology). In contrast, CN and SMC individuals with amyloid accumulation are subjects of ongoing debate, with no clear consensus on whether they represent a prodromal stage of AD or individuals at higher risk of developing the disease but still may never develop the disease (Frisoni et al., 2019; Jack, 2020). Table 1 shows the demographic information of the cohort used in this work.

**Table 1.** Demographic information of the A*β* cohort patients

Status	# of subjects	Age	Sex (M/F)	MMSE	A*β*_42_	CN	SMC	EMCI	LMCI	AD
A*β*−	185	71.8 ± 7.1	68/117	28.9 ± 1.5	1684.3 ± 601.2	69	75	41	–	–
A*β*+	133	74.5 ± 7.5	71/62	25.3 ± 4.3	607.5 ± 189.3	–	–	53	53	27

The MRI images for the considered cohort were collected as follows: (a) T1-weighted sMRI: TE/TR = shortest, inversion time = 900 ms, FOV = 256 × 256 mm^2^, 1-mm isotropic resolution, slices = 176–211; (b) rs-fMRI: TE/TR = 30/3000 ms, FOV = 220 × 220 × 163 mm^3^, 3.4-mm isotropic resolution, 200 volumes in almost all subjects, with minimal variations (e.g., 195–197) in a small subset; (c) single-shell dMRI: TE/TR = 56/7200 ms, FOV = 232 × 232 × 160 mm^3^, 2-mm isotropic resolution, *b* = 0 and 1000 s/mm^2^.

### Preprocessing and Feature Extraction

The sMRI preprocessing included tissue segmentation of GM, WM, and CSF with the modulated normalization algorithm in the Statistical Parametric Mapping toolbox (SPM12; https://www.fil.ion.ucl.ac.uk/spm/). This work used GM volumes smoothed with a Gaussian kernel (full width at half maximum (FWHM) = 6 mm). For QC, images that had a low correlation with individual- and/or group-level masks were discarded, which involved correlating data at three levels: the entire image, the top 20 slices, and the bottom 20 slices. The full preprocessed GM volume was input to the sMRI channel of the neural network (NN), resulting in an input size of 121 × 145 × 121 for each subject.

Adhering to the process proposed in Du et al. (2020), the rs-fMRI data were preprocessed with SPM12 including rigid body motion correction, removal of scans with high head motion parameters (> 3° of rotations and > 3 mm in translations), slice-timing correction, warping to the standard MNI space using the EPI template, resampling to 3 mm^3^ isotropic voxels, and smoothing with a Gaussian kernel (FWHM = 6 mm). QC was the same as for sMRI, correlating the data at three levels: the entire image, the top five slices, and the bottom five slices. Fifty-three maximally independent components (ICs) covering the whole brain were extracted using spatially constrained independent component analysis (ICA) with the Neuromark_fMRI_1.0 template (available in the GIFT software; https://trendscenter.org/software/gift). The ICs were divided into seven RSNs: the (a) subcortical (SuC), (b) auditory, (c) sensorimotor (SM), (d) visual (VI), (e) cognitive-control (CC), (f) DM, and (g) cerebellar (CB) networks. For each subject, the Pearson correlation between IC time courses was computed, resulting in a 53 × 53 static functional network connectivity (FNC) matrix, where FNC is the network analog of FC in that the time courses represent weighted partially overlapping whole brain patterns. Finally, each FNC matrix was converted into a complete, undirected, and weighted graph. The edges’ weights correspond to the FNC correlation values, considering both positive and negative values, while the values of the 53 nodes (i.e., ICs) were initialized at a value of one in order to force the network to learn a latent representation based only on the connectivity information. This FNC-based graph was the input to the rs-fMRI channel.

The dMRI volumes were preprocessed via brain extraction followed by Eddy currents correction (FSL 6.0; https://fsl.fmrib.ox.ac.uk/). The data were then denoised using local principal component analysis via empirical thresholds relying on the Python *dipy* library. Subsequently, nonlinear registration to the MNI space was applied to correct for EPI-induced currents. QC was performed during preprocessing by visual inspection of images before and after registration. MRtrix 3.0 (https://www.mrtrix.org/) was used to derive an anatomically constrained probabilistic tractography (2 million streamlines, step = 0.3 mm, maximum length = 300 mm, and backtracking) filtered with SIFT2 (Smith, Tournier, Calamante, & Connelly, 2015). Subject-specific brain parcellations from T1-weighted images were derived using FreeSurfer (https://surfer.nmr.mgh.harvard.edu/) and used as regions of interest (ROIs) in the SC calculation. The SC matrix was built by counting the number of streamlines connecting all pairs of regions from the Desikan-Killiany (Desikan et al., 2006) structural atlas, ignoring self-connections. Similarly to rs-fMRI, each SC matrix was converted into a complete, undirected, and weighted graph. In this case, the edges’ weights were defined as the number of streamlines between pairs of ROIs, and the values of the 84 nodes (i.e., anatomical ROIs) were initialized to 1. This SC-based graph was the input to the dMRI channel.

### Framework Architecture

**Figure 1.**
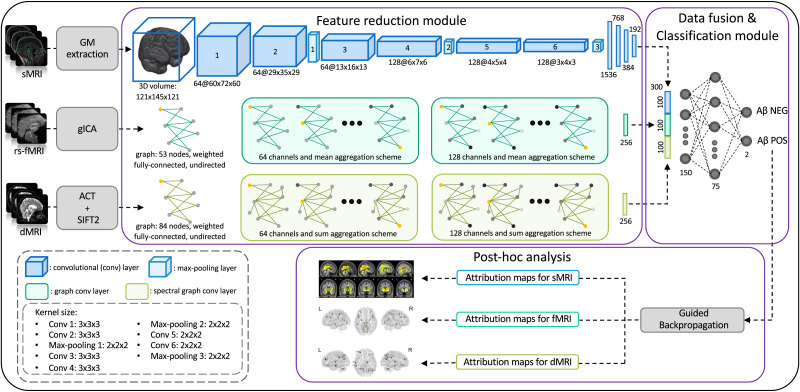
Schematic representation of the proposed framework. The model takes as input three MRI neuroimaging modalities: sMRI 3D volumes, rs-fMRI functional graph, and dMRI structural graph. The DL architecture is composed of two modules: (a) a feature reduction module, where the input data are transformed in their latent representations, and (b) a data fusion and classification module, where the latent feature of each modality are concatenated together and, finally, they are classified using a MLP.

The proposed framework is shown in Figure 1. In detail, the DL architecture used for the classification of A*β* status (positive/negative) has two modules: (a) a *feature reduction module* that actuates feature reduction using three different NNs to transform the input data into corresponding latent representations and (b) a *data fusion and classification module* that concatenates the latent representations of each modality and uses a multilayer perceptron (MLP) that takes the fused latent features as input for the final classification. Finally, a post hoc explainability analysis was performed on the correctly classified subjects to highlight the feature contributions to the classification task. In the following paragraphs, the two modules are further described.

#### Feature reduction module.

Three different NNs form the *feature reduction module*. Each NN extracts a latent representation of one modality, resulting in a latent vector of 100 features. The 3D sMRI volumes were analyzed using a 3D-CNN with six convolutional layers and three max pooling layers, completed with four dense layers. Conversely, both FNC and SC graphs were analyzed using two different GCNs. These models update the representation of each node, aggregating neighbor information iteratively in each layer through the message-passing scheme. The rs-fMRI channel was analyzed using a GCN with two graph convolutional layers proposed by Morris et al. (2019) that were followed by a dense layer. This type of convolutional layer is a powerful operator that integrates both high- and low-level structures along with their relationships into a single graph (Morris et al., 2019). The dMRI channel was analyzed with a different GCN with two Chebyshev spectral graph convolutional layers proposed by Defferrard, Bresson, and Vandergheynst (2016) followed by a dense layer. Spectral convolutional layers are high-performance layers that have been used effectively on irregular graphs (Parisot et al., 2018). These two architectures were chosen on an empirical basis as those leading to the best unimodal classification accuracy.

#### Data fusion and classification module.

The *data fusion and classification module* consists of a fusion layer and a classifier. The fusion layer concatenates the latent features extracted from the three channels, resulting in a vector of 300 features that incorporate information from all three modalities for each subject. Lastly, the latent vector was used as input for the final MLP classifier, composed of three dense layers. The convolutional and dense layers in this framework used a rectified linear unit (ReLU) activation function, except for the last layer, which used a softmax activation function to obtain the classification probabilities for each class.

### Training Scheme and Evaluation

The model was trained with stratified five fold cross-validation on the entire cohort to investigate its generalizability across individuals, and a hyperparameter search was performed empirically through a grid search procedure to maximize the average validation accuracy. To this end, different combinations were tested for the hyperparameters (batch size, learning rate, number of epochs, and regularization parameter). Additionally, different numbers of hidden layers for CNN, GCNs, and classifier were considered, also changing the number of channels for the convolutional layers and the number of neurons in the dense layers. The mini-batch strategy (with 16 subjects per batch) was finally adopted. The Adam optimizer (learning rate: 0.00001) was used to update the entire multimodal architecture. L2 regularization (weight decay: 0.0001) was applied to reduce overfitting. Weighted cross entropy was used as the loss function. The model was trained for 200 epochs.

Performance was evaluated using the mean evaluation accuracy, precision, recall, and F1 score over the five folds.

For the sake of comparison, we also tested the unimodal models with the corresponding MRI data, where the architectures were the same as the different branches of the multimodal framework (i.e., 3D-CNN for sMRI, and GCN for both rs-fMRI and dMRI).

## POST HOC ANALYSIS

### Guided Backpropagation

The post hoc XAI analysis was conducted using guided backpropagation (GBP) (Springenberg, Dosovitskiy, Brox, & Riedmiller, 2014). GBP uses the model gradients to extract the feature contribution maps with the same shape as the input data. It belongs to the “modified backpropagation” class of XAI methods in which the backward flow of gradients is modified with ReLU activation (Rahman, Calhoun, & Plis, 2023), setting the negative gradients to 0 and only allowing nonnegative gradients to be backpropagated. This approach enables the visualization of which input features activated the neurons and most contributed to the final prediction.

### Contribution Maps and Statistical Analysis

The attribution maps were extracted for the correctly classified A*β*-positive subjects. The average A*β*-positive subject attribution map was derived for identifying the most important features. To evaluate the sMRI GBP contribution maps, the Harvard-Oxford (Desikan et al., 2006) and the probabilistic CB (Diedrichsen, Balsters, Flavell, Cussans, & Ramnani, 2009) atlases from FSL were employed to define 56 different ROIs, including cortical, subcortical, and cerebellum regions. The sum of GBP attributions inside each ROI was calculated for the sMRI and weighted to account for the volume of each specific region. Conversely, the GBP attribution of each node for both rs-fMRI and dMRI was extracted directly from the two GCNs. Then, considering the average map for each modality, the percentage of explanation for each region/node was computed over the total contribution within and across modalities. The top 10 ROIs (sMRI) and nodes (rs-fMRI and dMRI) with the highest percentage of GBP contribution were selected for further investigation.

Subsequent statistical analyses were performed on the original data for all correctly classified subjects. For the sMRI, the mean values of the top 10 ROIs resulting from the XAI analysis were extracted from the input GM volumes and used as features for the statistical analysis. For the rs-fMRI and dMRI, graph-based measures were first derived from the full connectivity matrices in order to have a summary measure per node, and then only the top 10 nodes were retained for both rs-fMRI and dMRI for statistical analysis. In particular, the node strength was computed for the FNC matrices. This is defined as the sum of the weights of the edges connected with a given node, where in absolute terms, higher values mean more important nodes. Betweenness centrality was calculated for SC matrices, representing the fraction of all shortest paths in the SC matrix that contains the node under analysis, where the shortest path is the shortest sequence of nodes between node *i* and node *j*. As the sparsity of the SC matrix could limit the interpretation of the node strength results for the dMRI data, we preferred to rely on a centrality measure for this analysis. In this case, nodes that belong to more paths likely play a pivotal role in the propagation of the information inside the network. These two metrics were computed using the Brain Connectivity Toolbox (Rubinov & Sporns, 2010) in MATLAB.

Mann-Whitney tests were then used to compare the values of all these features for the top 10 ROIs/nodes between A*β*-positive and A*β*-negative correctly classified individuals. Finally, false discovery rate (FDR) correction for multiple comparisons was applied.

## RESULTS

### Classification Performance

The proposed framework for the classification of A*β* status achieved a mean ± standard accuracy, precision, recall, and F1 score of 0.762 ± 0.04, 0.694 ± 0.05, 0.774 ± 0.10, and 0.727 ± 0.05, respectively, across the evaluation folds.

The single networks that composed the multimodal framework along with the corresponding input data were also tested in the same classification task. Table 2 shows the performance comparisons for the multimodal and the three unimodal models. Results highlight how the multimodal pipeline is able to outperform the unimodal models in terms of accuracy, recall, and F1 score in the same classification task.

**Table 2.** Classification performance of the proposed multimodal framework with respect to the unimodal models for sMRI, rs-fMRI, and dMRI for the same classification task

Model	ACC	PRE	REC	F1
Multimodal	**0.762 ± 0.04**	0.694 ± 0.05	**0.774 ± 0.10**	**0.727 ± 0.05**
Unimodal sMRI	0.750 ± 0.06	**0.721 ± 0.06**	0.672 ± 0.16	0.683 ± 0.09
Unimodal fMRI	0.593 ± 0.05	0.423 ± 0.23	0.303 ± 0.21	0.338 ± 0.19
Unimodal dMRI	0.603 ± 0.04	0.419 ± 0.25	0.311 ± 0.28	0.322 ± 0.23

ACC = accuracy; REC = recall; PRE = precision.

### GBP-Based Attribution Maps

The evaluation set in the fold with the highest evaluation accuracy was used for post hoc analysis. Importantly, in the analyzed fold, the evaluation and training sets were disjoint.

**Figure 2.**
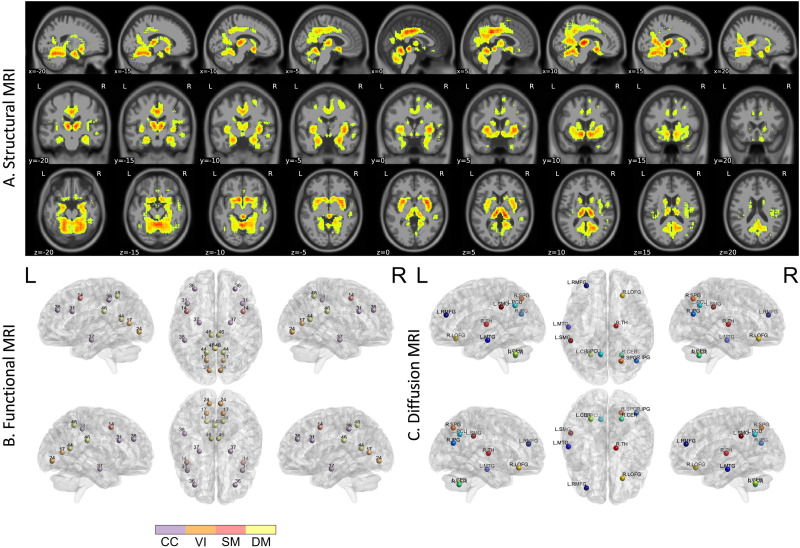
GBP-based attributions for the A*β*-positive mean subject derived from the correctly classified individuals overlaid to the MNI152 template, where (A) sagittal, coronal, and axial views for the average sMRI GBP map where only the attributions exceeding the 96th percentile are shown, highlighting both cortical and subcortical regions; (B) the 10 most important nodes (ICs) from the rs-fMRI data, representing mainly the DM and CC brain networks; and (C) the 10 most important nodes (ROIs) from the dMRI data, involving both cortical and subcortical regions in both hemispheres, also including the cerebellum.

Figure 2A shows the sMRI GBP attribution map for the A*β* mean positive subject, overlaid to the MNI template. Qualitatively, mainly subcortical regions (e.g., hippocampus, thalamus) were relevant to the final classification along with a few cortical areas.

Figure 2B shows the 10 most important nodes for the rs-fMRI modality. All displayed nodes (ICs) are bihemispheric, except for the left inferior parietal lobule (IC38) node, which is mainly in the left hemisphere. The most important nodes belong to the DM (three nodes), CC (four nodes), VI (two nodes), and SM (one node) networks.

Finally, Figure 2C shows the 10 most important dMRI nodes (ROIs). Subcortical regions (thalamus, cerebellum) along with areas in the frontal, temporal, and parietal lobes were identified.

### Feature Relevance and Statistical Analysis

**Figure 3.**
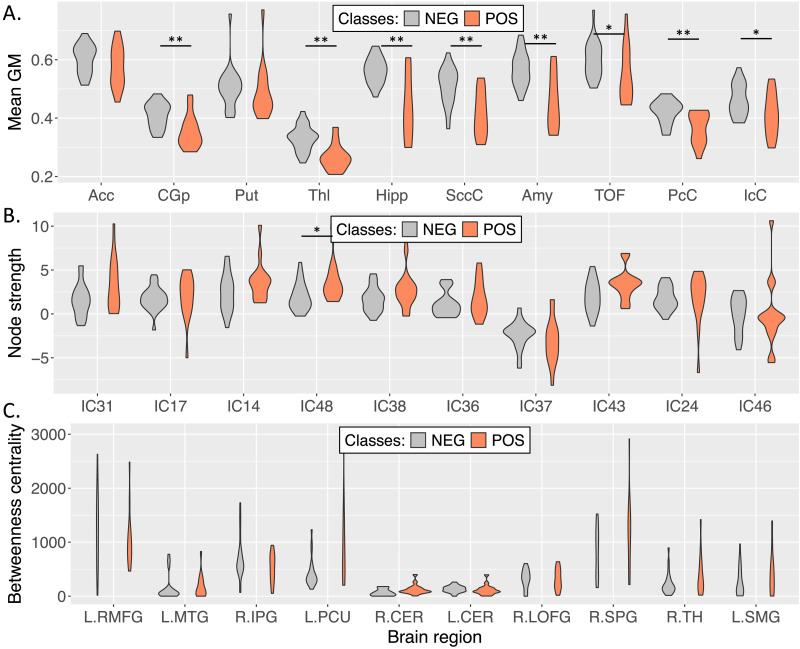
Distributions of the input data for the 10 most important brain regions/nodes considered in the statistical analysis for each modality. (A) Between-subject distribution of the regional (mean) GM volumes for sMRI, (B) between-subjects distribution of the node strength values for rs-fMRI, and (C) between-subject distribution of the betweenness centrality values for dMRI.

Figure 3A shows the violin plots for the top 10 sMRI features, while Table 3A shows the corresponding percentages of mean GBP contributions across A*β*-positive correctly classified subjects for the same regions, weighted by their volumes. The table also reports the *p*-values and FDR-corrected *p*-values of the statistical tests performed on the input features. We identified significant differences between classes after FDR correction in the *precuneus, cingulate gyrus* (posterior division), *thalamus, hippocampus, supracalcarine* and *intracalcarine cortices, amygdala*, and *temporal occipital fusiform cortex*, showing an overall comparison direction of *NEG* > *POS*.

**Table 3.** Percentage of GBP explanations (for the A*β* mean positive subject) and the results from the statistical tests for the top 10 brain regions derived from (A) sMRI, (B) rs-fMRI, and (C) dMRI

(A) sMRI					
Region		Percentage (%)	*p*-value	FDR adj. *p*-value	Comparison direction
Accumbens^†^ (Acc)		6.59%	0.20402	0.22669	n.s.
Cingulate gyrus, posterior^†^ (CGp)		4.91%	**0.00182**	**0.00304**	NEG > POS
Putamen^†^ (Put)		4.59%	0.26024	0.26024	n.s.
Thalamus^†^ (Thl)		4.09%	**0.00016**	**0.00146**	NEG > POS
Hippocampus^†^ (Hipp)		3.95%	**0.00073**	**0.00146**	NEG > POS
Supracalcarine cortex^†^ (SccC)		3.81%	**0.00043**	**0.00146**	NEG > POS
Amygdala^†^ (Amy)		3.45%	**0.00066**	**0.00146**	NEG > POS
Temporal occipital fusiform cortex^†^ (TOF)		3.22%	**0.01793**	**0.02561**	NEG > POS
Precuneus cortex^†^ (PcC)		3.07%	**0.00066**	**0.00146**	NEG > POS
Intracalcarine cortex^†^ (IcC)		2.83%	**0.03509**	**0.04386**	NEG > POS

(B) rs-fMRI					
Region	RSN	Percentage (%)	*p*-value	FDR adj. *p*-value	Comparison direction
Middle frontal gyrus∗ (IC31)	CC	9.58%	0.10597	0.21194	NEG > POS
Calcarine gyrus (IC17)	VI	6.79%	0.34078	0.42597	n.s.
Precentral gyrus (IC14)	SM	6.31%	**0.04965**	0.16549	NEG > POS
Precuneus∗ (IC48)	DM	6.08%	**0.00389**	**0.03892**	NEG > POS
Left inferior parietal lobule (IC38)	CC	4.20%	0.07349	0.18372	NEG > POS
Middle frontal gyrus∗ (IC36)	CC	4.01%	0.32634	0.42597	n.s.
Hippocampus (IC37)	CC	3.76%	0.23658	0.38431	n.s.
Precuneus∗ (IC43)	DM	3.65%	**0.02623**	0.13115	NEG > POS
Lingual gyrus (IC24)	VI	3.23%	0.38648	0.42942	n.s.
Posterior cingulate cortex (IC46)	DM	2.94%	0.50672	0.50672	n.s.

(C) dMRI					
Region		Percentage (%)	*p*-value	FDR adj. *p*-value	Comparison direction
Rostral middle frontal LH (L.RMFG)		39.140%	0.35559	0.44448	n.s.
Middle temporal LH (L.MTG)		9.280%	0.49705	0.55227	n.s.
Inferior parietal RH (R.IPG)		6.550%	0.20919	0.34865	n.s.
Precuneus LH (L.PCU)		5.460%	**0.01470**	0.14700	NEG > POS
Cerebellum RH (R.CER)		4.480%	**0.03887**	0.19433	NEG > POS
Cerebellum LH (L.CER)		3.940%	0.14061	0.28187	n.s.
Lateral orbito frontal RH (R.LOFG)		3.780%	0.96545	0.96545	n.s.
Superior parietal RH (R.SPG)		3.760%	0.07825	0.26084	NEG > POS
Thalamus RH (R.TH)		3.300%	0.14094	0.28187	n.s.
Supra marginal LH (L.SMG)		3.290%	0.31926	0.44448	n.s.

The term n.s. means not statistically significant, the sign^†^ means top 10 regions across the three modalities, while the sign* means ICs with different spatial locations in rs-fMRI, LH/RH means left/right hemisphere, respectively, in dMRI, NEG/POS mean negative and positive groups, respectively, and FDR means false discovery rate". **Boldface** means that the *p*-value is statistically significant.

Figure 3B shows violin plots representing the graph node strength for the top 10 rs-fMRI nodes, while Table 3B shows the mean percentages of GBP contributions extracted from the same nodes (ICs) across A*β*-positive patients. After FDR correction, the *precuneus* (IC48) was the only region with significant differences (*NEG* < *POS*). Before correction, the *precuneus* component (IC43) and *precentral gyrus* (IC14) were also significant with *NEG* < *POS*, while the *left inferior parietal lobule* (IC38) exhibited a trend toward uncorrected statistical significance (*p* < 0.10, with *NEG* < *POS*).

Figure 3C shows the violin plots representing the graph betweenness centrality for the top 10 dMRI nodes, and Table 3C shows the mean percentages of GBP contributions for the same nodes across A*β*-positive correctly classified subject. The *precuneus* left hemisphere (LH) and *cerebellum* right hemisphere (RH) were significant when considering uncorrected *p*-values (*NEG* < *POS*), but none of the results survived multiple comparisons correction. Moreover, the *right superior parietal gyrus* showed a trend toward significance (*p* = 0.07825, *NEG* < *POS*).

We also computed the percentage of contribution of each region for the three modalities combined, retrieving the 10 most important areas (marked with^†^ in Table 3A–C). All the 10 most important brain regions belonged to the sMRI modality (Table 3A).

## DISCUSSION

In this work, we proposed a multimodal data fusion framework that integrates multiple MRI techniques (sMRI, rs-fMRI, and dMRI) for the classification of A*β* status in an unbalanced cohort of subjects. A post hoc XAI analysis and evaluation was performed using GBP to assess feature importance, complemented by statistical analyses on the so-identified input features for validation via post hoc plausibility assessment.

### Classification Performance

Results showed that the multimodal framework outperformed single-modality models in terms of classification performance, particularly for rs-fMRI and dMRI, while the sMRI achieved performance close to the multimodal one, but with higher variance across the evaluation folds. This provides evidence of the added value brought by multimodality approaches in terms of classification accuracy while injecting complementary information, shedding light on the underlying neurophysiological mechanisms, which is particularly relevant for the study of complex neurodegenerative diseases influenced by multidomain factors.

Table 4 shows the performance of state-of-the-art (SOA) works for the classification of A*β*-positive versus A*β*-negative subjects. Of note, current SOA works use multiple datasets and approaches for addressing this task. It goes without saying that the lack of a common reference dataset inherently limits the relevance of performance comparison. However, reaching good accuracy and outperforming the SOA does not exhaust the contribution of the proposed approach, whose potential lies in decoding the neurophysiological changes induced by the A*β* status as captured by the considered MRI modalities.

**Table 4.** Comparison of the proposed model with other SOA approaches for the classification of A*β*-positive (POS) versus A*β*-negative (NEG) conditions

Authors	Modalities	Study cohort	Input data	Model	ACC	REC	PRE	F1
Kim et al. (2021)	A*β*/FDG-PET	738 POS, 815 NEG	3D FDG-PET	2.5D CNN	0.733^∗^–0.690^†^	0.678^∗^–0.768^†^	n.d.	0.709^∗^–0.712^†^
Lee et al. (2021)	A*β* PET	350 POS, 333 NEG	3D A*β* PET	Inception3D	0.954^∗^–0.767^†^	0.918^∗^–0.845^†^	n.d.	n.d.
				ResNet3D	0.920^∗^–0.671^†^	0.918^∗^–0.944^†^		
				VGG3D	0.977^∗^–0.870^†^	0.959^∗^–0.831^†^		
Ladefoged et al. (2023)	A*β* PET	POS, NEG n.d.	3D A*β* PET	3D-CNN	0.980^∗^–0.990^†^	0.980^∗^–0.990^†^	n.d.	0.980^∗^–0.990^†^
		1309 + 224						
Rasi et al. (2024)	FDG-PET	185 POS, 116 NEG	FDG-PET-derived features	LASSO+GNB	0.924 (AUC)	n.d.	n.d.	n.d.
Yang et al. (2021)	sMRI	151 AD POS, 232 CN NEG	Hippocampus region	SDF-based NN	^1^0.772 ± 0.03	n.d.	n.d.	n.d.
		171 MCI POS, 171 MCI NEG			^2^0.592 ± 0.05			
Chattopadhyay et al. (2023)	sMRI	POS, NEG n.d.	3D volume	3D-CNN	0.760^†^	n.d.	n.d.	0.746
		459 CN, 67 MCI, 236 AD						
Proposed framework	sMRI, fMRI, dMRI	133 POS, 185 NEG	GM volume FNC graph, SC graph	Multimodal DL model	0.762 ± 0.04^∗^	0.774 ± 0.10^∗^	0.694 ± 0.05^∗^	0.727 ± 0.05^∗^

^1^ = AD A*β*+ vs. CN A*β*−;^2^ = MCI A*β*+ vs. MCI A*β*−; * = validation set;^†^ = test set; n.d. = not declared

Most of the SOA works relied on PET scans to address this task, for example, Kim et al. (2021) developed a CNN model that takes the different views (axial, coronal, and sagittal) of a 3D volume as input, while Lee et al. (2021) used three different well-known 3D-CNN (Inception3D, ResNet3D, and VGG3D) models to address this task using the full 3D volume of PET scans. They achieved an accuracy of around 0.710 on average in the test set, with a maximum accuracy of 0.870 by Lee et al. (2021) using the VGG3D architecture. In the same way, Ladefoged et al. (2023) employed a 3D-CNN to identify the A*β* status, reporting both the average validation accuracy and also the accuracy on a hold-out test set (ADNI data) using the ensemble method created with the best models across the different folds, achieving accuracies of around 0.980. Recently, Rasi et al. (2024) employed different combinations of feature selection methods and classifiers in order to analyze FDG-PET-derived features. They achieved an AUC of 0.924, employing LASSO with the GNB model. Only a few works used MRI modalities to classify A*β*-positive and A*β-*negative subjects. Yang et al. (2021) focused on the hippocampus region only for detecting A*β* positivity, testing the network on two different classification tasks: AD A*β* positive versus CN A*β* negative, and MCI A*β* positive versus MCI A*β* negative, achieving an accuracy of 0.772 ± 0.03 and 0.592 ± 0.05 in the first and second tasks, respectively. Lastly, Chattopadhyay et al. (2023) implemented a 3D-CNN for detecting A*β* status from sMRI images defining the A*β* status of the CN, MCI, and AD patients considering the PET cortical standardized uptake value ratio reaching an accuracy of 0.760.

Comparing the proposed method with these six works, we outperformed the method that relied on 3D FDG-PET proposed by Kim et al. (2021) and the performance of Chattopadhyay et al. (2023). Our study achieved competitive performance in the validation set compared with Lee et al. (2021) using amyloid PET images, although our pipeline led to higher accuracy compared with the ResNet3D they used, and a similar accuracy to that in Yang et al. (2021) using the hippocampus region only. Besides providing competitive performance, our model allows to successfully integrate structural GM features extracted from a 3D-CNN along with FC and SC relying on two ad hoc GCNs. Notably, while a few studies have recently started to explore SC/FC in conjunction with GCNs in the AD classification task (J. Zhang et al., 2023; Y. Zhang, He, Chan, Teng, & Rajapakse, 2023), results are still limited, calling for further investigation in the AD continuum.

### Explainability Analysis

The post hoc analysis, performed on the correctly classified subjects, consisted of two steps: (a) extraction of GBP attribution maps for A*β* mean positive subjects as well as the corresponding percentage of contribution for each brain region, both within and between modalities, and (b) statistical analysis on the input features of the 10 most important regions/nodes identified by GBP.

The analysis of the sMRI attribution maps revealed that multiple brain regions involved in AD neurodegeneration could be identified. These regions had a higher percentage of contribution relative to the others and belonged to subcortical areas (hippocampus, thalamus, putamen, accumbens, and amygdala) and temporal/occipital/parietal areas (posterior cingulate gyrus, precuneus cortex, supracalcarine and intracalcarine cortices, and the temporal occipital fusiform cortex). The hippocampus, in particular, is a well-known biomarker for AD that is subject to high levels of atrophy. Some studies have suggested that this atrophy is attributable to the deposition of A*β* plaques (Cantero, Iglesias, Van Leemput, & Atienza, 2016). An important region that is connected with the hippocampus is the cingulate gyrus, which showed a strong reduction of GM in AD patients (Green et al., 2023; Jones et al., 2006). In a clinical study performed by M. S. Kang et al. (2021), significant associations were detected between A*β* accumulation and GM atrophy in the hippocampus and posterior cingulate gyrus for the MCI and AD A*β*-positive subjects. Some works also linked the nucleus accumbens region to AD pathology and progression. Evidence of GM loss and alteration of nucleus accumbens in MCI and AD, with respect to the CN, had previously been highlighted (Nie et al., 2017; Yi et al., 2016). Additionally, Guo et al. (2022) showed how A*β* oligomers in nucleus accumbens can promote synaptic loss and motivation deficits in AD. Other subcortical structures, like the thalamus and putamen, have a high atrophy rate in clinical patients relative to healthy individuals (de Jong et al., 2008; M. S. Kang et al., 2021), probably due to A*β* deposition. A previous study highlighted an increase in the standardized uptake value ratio (derived from florbetapir PET) in both putamen and thalamus in the preclinical stages of AD (Edmonds et al., 2016). Following the statistical analysis performed on the input GM volumes, significant differences were detected in the hippocampus, posterior cingulate gyrus, amygdala, thalamus, and precuneus, highlighting a considerable decrease of GM in the A*β*-positive subjects. These findings were consistent with preexisting literature. Additionally, Thal, Rüb, Orantes, and Braak (2002) highlighted other regions (that we also found to be relevant) involved in the different phases of A*β* deposition (i.e., proisocortex, allocortical areas, diencephalic nuclei, and striatum).

For the rs-fMRI, the brain regions (ICs) that most contributed to the final classification resided in the DM and CC networks, along with three regions for VI and SM networks (two and one, respectively). The DM network is involved in memory, self-knowledge, and thinking, and it is highly related to AD (Green et al., 2023; Pini et al., 2021). Previous studies also suggested that DMN is associated with the presence of A*β* plaques (Matthews, Filippini, & Douaud, 2013). The CC network is associated with selective attention, working memory, and stimulus-response mapping (Miller, 2000; Sendi et al., 2023). The statistical analysis, performed on the node strength values, detected one region with a significant difference between the two groups after FDR correction and three regions before (uncorrected *p*-values). One more region, the left inferior parietal lobule, exhibited a trend toward significance. Specifically, the A*β*-positive class had a significantly increased node strength in the precentral gyrus (IC14, SM network), which was consistent with the findings of Guzmán-Vélez et al. (2022) and Duan et al. (2017). The precuneus regions (IC43 and IC48, DM network) were also significantly different between groups with increased strength in the positive group. As in other works (Celone et al., 2006; Dadario & Sughrue, 2023), this suggested that the precuneus plays a key role and undergoes pathological changes related to AD. Previous works also identified the posterior DM (the precuneus and posterior cingulate cortex) (Palmqvist et al., 2017) and parts of the CC network (i.e., the middle frontal gyrus and hippocampus) (Canuet et al., 2015; Pereira et al., 2019) as strongly affected by A*β* deposition.

Lastly, in the dMRI channel, GBP identified multiple cortical and subcortical regions as relevant to the outcome of our model. The cerebellum resulted to be important for the model. Of note, the rostral middle frontal gyrus was assigned a high percentage of contribution relative to the other regions. Additionally, the middle temporal gyrus, precuneus, thalamus, and superior parietal regions showed high importance relative to the other 84 regions. After FDR correction, no region had statistically significant group differences in betweenness centrality values. On the other hand, based upon uncorrected *p*-values, significant differences (*p* < 0.05) were detected in the precuneus and cerebellum (right hemisphere), while the superior parietal region was close to significance (*p* = 0.07825). Regarding the two nodes with significant uncorrected differences, the precuneus, which is also identified in the rs-fMRI, is an important hub for functional operations because it is highly connected with other regions by both short- and long-range WM fibers (Dadario & Sughrue, 2023). On the other hand, different studies highlighted how the cerebellum is subject to increase A*β* deposition in AD pathology (Braak, Braak, Bohl, & Lang, 1989; Catafau et al., 2016) at different stages (Stage 3 in Braak & Braak, 1998, and Stage 5 in Thal et al., 2002) relative to normally aged subjects. Widespread structural alterations in WM tracts connecting cortical and subcortical regions in both hemispheres were detected between A*β*-negative CN and A*β*-positive preclinical AD patients by Molinuevo et al. (2014).

When combining the explanations of all channels, we found that sMRI most contributed to the final prediction. This was expected as we initially assumed that the effects of A*β* plaques would predominantly impact GM volumes.

### Common Regions Across Modalities

Multiple brain regions were identified as important by GBP across all three channels. Specifically, the *precuneus* was highlighted in all three modalities. It was one of the most interesting regions as it plays a pivotal role in the transmission of functional information due to the high concentration of WM tracts linking the precuneus with other brain areas. Our finding of decreased GM and increased functional node strength for the region in A*β*-positive subjects could be related to the functional compensation effects of AD and deserves further investigation (Celone et al., 2006; Dadario & Sughrue, 2023). Additionally, the *cingulate gyrus* (*posterior division*), *calcarine gyrus*, and *hippocampus* were important in both sMRI and rs-fMRI. Only the *thalamus* was relevant to both sMRI and dMRI, and the *middle frontal gyrus* was the only area important to both rs-fMRI and dMRI. Interestingly, the *middle frontal gyrus* was a region highlighted in both rs-fMRI and dMRI, but not in the sMRI. This further underlines the importance of integrating different (and complementary) modalities to provide a complete picture of the complex pathological mechanisms.

### Main Contributions and Outcomes

We proposed a multimodal and explainable neuroimaging DL model for the classification of A*β*-positive or A*β*-negative status. The main contribution of this work is a framework that (a) successfully integrates volumetric features and connectivity information from sMRI, rs-fMRI, and dMRI data, extracted from one 3D-CNN and two GCNs, respectively; (b) obtains competitive classification performance (ACC = 0.762 ± 0.04) relative to the SOA; and (c) provides insight into the brain regions that most contribute to final model outcome using a GBP-based post hoc analysis. Finally, we evaluated the XAI outcomes by assessing the discriminative power of the selected input features across classes.

With these analyses, we identified multiple brain regions that could be altered by A*β* plaque deposition, resulting in atrophy, functional changes, and WM connectivity changes relative to the A*β*-negative class. Furthermore, the analysis identified common regions across modalities, including the *precuneus, hippocampus, thalamus, cingulate gyrus, calcarine gyrus*, and *middle frontal gyrus*, strengthing the evidence of their involvement in this pathological process.

Our findings highlight the utility of MRI for studying the possible effects of A*β* deposition and the importance of integrating complementary information to enable a better understanding of the differences related to amyloid status. However, we acknowledge that the two main limitations of this work are the limited number of subjects in the dataset, having both A*β* information and all the considered MRI modalities, and the unbalanced cohort including CN, SMC, and EMCI in the A*β*-negative group and EMCI, LMCI, and AD in the A*β*-positive one. Hence, the outcomes could be guided by those factors that were influenced by the amyloid accumulation as captured by the specific modality. In consequence, the GM branch captured atrophy, while the SC and FC branches captured the respective amyloid-induced connectivity modulations, leading to a combined effect due to A*β-* and dementia-related processes. Further studies are thus needed to assess the generalizability of the proposed multimodal framework to larger and possibly employing balanced datasets, relying on study cohorts composed of all clinical classes (CN, SMC, EMCI, LMCI, and AD) in both groups analyzed, A*β*-negative and A*β*-positive individuals, in order to disentangle the contributions of the different factors and, hence, having the outcomes only related to A*β*.

### Conclusion

In this work, we presented a multimodal and explainable DL-based framework for the classification of A*β* status, exploiting anatomical and connectivity MRI-based information. The application of GBP enabled the identification of the regions most important to the final model predictions, some of which were common across modalities, (e.g., the precuneus, hippocampus, thalamus, cingulate gyrus, calcarine gyrus, and middle frontal gyrus). Our study demonstrates the potential viability of noninvasive MRI-based detection of A*β* status involving multimodal data, paving the way for further research in this direction.

## ACKNOWLEDGMENTS

Data collection and sharing for this project was funded by the ADNI (National Institutes of Health Grant U01 AG024904) and DOD ADNI (Department of Defense award number W81XWH-12-2-0012). ADNI is funded by the National Institute on Aging, the National Institute of Biomedical Imaging and Bioengineering, and through generous contributions from the following: AbbVie, Alzheimer’s Association; Alzheimer’s Drug Discovery Foundation; Araclon Biotech; BioClinica, Inc.; Biogen; Bristol-Myers Squibb Company; CereSpir, Inc.; Cogstate; Eisai, Inc.; Elan Pharmaceuticals, Inc.; Eli Lilly and Company; EuroImmun; F. Hoffmann-La Roche Ltd. and its affiliated company Genentech, Inc.; Fujirebio; GE Healthcare; IXICO Ltd.; Janssen Alzheimer Immunotherapy Research & Development, LLC.; Johnson & Johnson Pharmaceutical Research & Development, LLC.; Lumosity; Lundbeck; Merck & Co., Inc.; Meso Scale Diagnostics, LLC.; NeuroRx Research; Neurotrack Technologies; Novartis Pharmaceuticals Corporation; Pfizer, Inc.; Piramal Imaging; Servier; Takeda Pharmaceutical Company; and Transition Therapeutics. The Canadian Institutes of Health Research is providing funds to support ADNI clinical sites in Canada. Private sector contributions are facilitated by the Foundation for the National Institutes of Health (www.fnih.org). The grantee organization is the Northern California Institute for Research and Education, and the study is coordinated by the Alzheimer’s Therapeutic Research Institute at the University of Southern California. ADNI data are disseminated by the Laboratory for NeuroImaging at the University of Southern California.

This study was funded by the National Institutes of Health Grant RF1AG063153 and National Science Foundation Grant 2112455, as well as Fondazione CariVerona (EDIPO project, num. 2018.0855.2019) and MIUR D.M. 737/2021 “AI4Health: empowering neurosciences with eXplainable AI methods.”

## AUTHOR CONTRIBUTIONS

Giorgio Dolci: Conceptualization; Data curation; Formal analysis; Investigation; Methodology; Software; Writing – original draft. Charles A. Ellis: Methodology; Writing – original draft. Federica Cruciani: Data curation; Methodology; Writing – review & editing. Lorenza Brusini: Data curation; Writing – review & editing. Anees Abrol: Data curation; Writing – review & editing. Ilaria Boscolo Galazzo: Data curation; Investigation; Writing – review & editing. Gloria Menegaz: Conceptualization; Funding acquisition; Supervision; Writing – review & editing. Vince D. Calhoun: Conceptualization; Funding acquisition; Supervision; Writing – review & editing.

## FUNDING INFORMATION

Vince D. Calhoun, National Institute of Health, Award ID: RF1AG063153. Vince D. Calhoun, National Science Foundation (https://dx.doi.org/10.13039/100000001), Award ID: 2112455. Gloria Menegaz, Fondazione CariVerona, Award ID: 2018.0855.2019. Gloria Menegaz, MIUR, Award ID: D.M. 737/2021 “AI4Health: Empowering neurosciences with eXplainable AI methods.”

## ADDITIONAL INFORMATION

The data used in this work were collected by ADNI (https://adni.loni.usc.edu/), and they are publically available after requested on the ADNI website.

## ETHICAL STANDARD

The data used in this work were acquired by ADNI (https://adni.loni.usc.edu/). Information regarding the ethical standard and informed consent of ADNI 3 Protocol are available at the following link: https://adni.loni.usc.edu/wp-content/themes/freshnews-dev-v2/documents/clinical/ADNI3_Protocol.pdf.

## TECHNICAL TERMS

Amyloid-*β* plaques: Deposition of amyloid-*β* proteins in the extracellular space.
Amyloid-*β* status: A subject considered positive or negative based on the amyloid-*β* level in the CSF.
Tractography: Method to *in vivo* detect and visualize white matter tracts in the brain.
SC: Structural connectivity matrix expressed as the number of streamlines between pairs of brain regions.
FC: Functional connectivity matrix expressed as Pearson correlation values between pairs of time courses.
CNN: Convolutional neuronal network represents a type of deep learning model widely used to analyze images.
GCN: Graph convolutional network is a type of deep learning algorithm used to analyze unstructured data, for example, graph.
XAI: EXplainable AI is a branch of AI with the scope to understand what AI algorithm has learned.
Graph: Complete, undirected, and weighted graph defined by nodes (brain regions), edges (link between nodes), and weights (FC/SC values).
Node: Element of a graph that represents a region in a brain atlas.
FNC: Functional network connectivity matrix expressed as Pearson correlation values between pairs of component/network time courses.
Edge: The link between two pairs of nodes with weighted equal to the correlation value between the two nodes.
A/T/N framework: A framework used to describe the amyloid-β, tau, and neurodegeneration in AD progression.
